# Ameliorative Effects of Honey, Propolis, Pollen, and Royal Jelly Mixture against Chronic Toxicity of Sumithion Insecticide in White Albino Rats

**DOI:** 10.3390/molecules25112633

**Published:** 2020-06-05

**Authors:** Atef M.K. Nassar, Yehia M.M. Salim, Khalid S.A. Eid, Hazem M. Shaheen, Abdullah A. Saati, Helal F. Hetta, Amr Elmistekawy, Gaber El-Saber Batiha

**Affiliations:** 1 Plant Protection Department, Faculty of Agriculture, Damanhour University, Damanhour 22511, AlBeheira, Egypt; yehia.salim@agr.dmu.edu.eg (Y.M.M.S.); khalid.eid@agr.dmu.edu.eg (K.S.A.E.); 2 Department of Pharmacology and Therapeutics, Faculty of Veterinary Medicine, Damanhour University, Damanhour 22511, AlBeheira, Egypt; dr_hazemshaheen3010@yahoo.com; 3 Department of Community Medicine & Pilgrims Healthcare, Faculty of Medicine, Umm Al-Qura University, Makkah 24382, Saudi Arabia; aaasaati@uqu.edu.sa; 4 Department of Medical Microbiology and Immunology, Faculty of Medicine, Assiut University, Assiut 71515, Egypt; helal.hetta@uc.edu; 5 Departmentof Internal Medicine, University of Cincinnati College of Medicine, Cincinnati, OH 45267, USA; 6 Department of Internal Medicine, Gastroenterology Division, Faculty of Medicine, Al-Azhar University, Cairo 11651, Egypt; drmistekawy@azhar.edu.eg

**Keywords:** sumithion, bee products, hematological, renal, hepatotoxicity, oxidative stress, AChE

## Abstract

Sumithion (Fenitrothion) (SUM) is an organophosphorus insecticide used to combat a wide variety of plant pests. Exposure to SUM causes significant toxicity to the brain, liver, kidney, and reproductive organs through, for example, binding to DNA, and it induces DNA damage, which ends with oxidative stress. Therefore, the present study aimed to examine the protective role of bee products: a mixture of honey, propolis, palm pollen, and royal jelly (HPPJ) against SUM-induced toxicity. Twenty-four male albino rats (*Rattus norvegicus*) were classified into four groups, each containing six rats: control (corn oil), SUM (85 mg/kg; 1/20 LD_50_), HPPJ, and SUM + HPPJ once daily for 28 consecutive days. Blood samples were gently collected in sterilized ethylenediaminetetraacetic acid (EDTA) tubes for blood picture analyses and tubes without anticoagulant for serum isolation. Serum was used for assays of enzymatic and biochemical characteristics. The results revealed that SUM increased the weights of the liver, kidney, and brain as well as the enzymatic activity of glutathione peroxidase (GP), serum superoxide dismutase (SOD), and glutathione-S-transferase (GST). Additionally, SUM significantly increased the activity of lactate dehydrogenase (LDH), alkaline phosphatase (ALP), and γ-glutamyltransferase (γ-GT) and glucose, uric acid, and creatinine contents, while decreasing the acetylcholine esterase (AChE) activity and total lipids and total protein content. Furthermore, because of the inclusion of phenolic, flavonoids, terpenoids, and sugars, the HPPJ mixture counteracted the hematological, renal, and hepatic toxicity of SUM exposure.

## 1. Introduction

The intensification of pesticides exhibits a prompt threat to human health, while the gradual build-up of pesticides in the environment and the human body cause serious toxic effects [1,2]. Organophosphorus pesticides (OPs) are readily used worldwide to control public health and agricultural pests [3] and have a significant potential health hazard due to their acute or chronic poisoning [4]. Sumithion (SUM, *O, O*-dimethyl-*O*-(3-methyl-4-nitrophenyl) phosphorothioate) is a contact-acting-OP insecticide that is used against mites and insects on vegetables, rice, cereals, and cotton [5]. SUM is distinguished by a rapid and severe absorption in the digestive tract and a preferable aggregation in the liver and blood [6]. Previous studies have shown that SUM causes remarkable changes in several organs such as the kidneys, brain, reproductive organs, and liver [5,6]. Additionally, many reports revealed that OPs bind to DNA [7]. In turn, the DNA damage triggers a machinery response like MAPK, NF-κB, and p53 mechanisms that control genes associated with the metabolism of reactive oxygen species (ROS) and Ca^+2^ apoptosis and homeostasis [8]. Therefore, Vanova et al. [9] hypothesized that OPs’ phosphorylate proteins/low molecular antioxidants had a pro-oxidative effect.

Moreover, many experiments have reported that OPs affect many vital organs, such as chronic OP toxicity, which may cause extreme liver cell injury [10]. Endogenous antioxidant status, essential trace elements, and liver enzymes are negatively affected by chronic rat intoxication with OPs [11]. Besides, hematological parameters were considered to be a rapid bioindicator of toxicity after prolonged exposure to OPs including malathion [12]. To lessen the toxicity of pesticides, several approaches were attempted, including the intake of medicinal herbs and/or their natural constituents [13]. Honey as a natural product and bee products (propolis, wax, pollen, royal jelly) have caught researchers’ attention as a supplementary and alternate therapy due to their antioxidant properties [14,15,16]. Moreover, they were used as substances for treating wounds, burns, cancer, cardiovascular, gastrointestinal tract, and neurodegenerative disorders [17,18,19]. Propolis was reported as having an ameliorative activity against chlorpyrifos and profenofos toxicity by increasing the immune capacity by significantly increasing the immunoglobulins [20]. Honey at a dose of 1.2 g/kg increased the activity and amount of antioxidants like vitamin C, β-carotene, uric acid, and glutathione reductase in healthy people [21].

However, bee products are known as possible sources of natural antioxidants, which can combat oxidative stress because of their phenolic compound (phenolic acids and flavonoids) content that expresses a scavenging activity of free radicals [22]. Additionally, these phenolics, sugars, proteins, carotenes, amino acids, Maillard reaction products, organic acids, and other minor compounds participate in the antioxidant effects of honey and other bee products [23]. The proposed mechanisms include the sequestration of free, superoxide, and hydroxyl radicals, metallic ion chelation, and hydrogen donation [24,25].Therefore, the current research was planned to a) assess the effects of SUM oral administration for 28 successive days on the hematological, renal, hepatic, and oxidative toxicity of male white albino rats, b) investigate the ameliorative role of a bee products-based mixture (honey, propolis, palm pollen, and royal jelly) against possible SUM toxic effects, and c) discriminate the chemical constituents of the mixture using the gas chromatography-mass spectroscopy (GC-MS) technique. 

## 2. Materials and Methods

### 2.1. Chemicals and Materials

Sumithion (*O, O*-dimethyl-*O*-(4-nitrophenyl) phosphorothioate, CAS 122–14–5, 99% purity) was purchased from Supelco Analytical (PA, USA), dissolved in corn oil and stored for future use. 5,5-dithiobis-2-nitrobenzoic acid (DTNB-Ellman’s reagent: Sigma order #D218200), bovine serum albumin (BSA: Sigma order #A2153-10G), acetylthiocholine iodide (ATChI; Sigma order #A5751-250MG), and brilliant (Coomassie) blue G-250 (Sigma order #B0770-5G) were purchased from Sigma-Aldrich through the local agent (AGITECH), Cairo, Egypt. Citrus bee honey and fresh royal jelly were collected from healthy honeybee colonies at an apiary located at Kafr El-Dawar district, El-Beheira Governorate, Egypt. Fresh palm pollen grains were collected from palm trees at Rashid district, El-Beheira Governorate. Refined propolis powder was obtained from the local market and ground into a fine powder. Then, bee products were mixed as a mixture of 22.5 g of citrus bee honey, 1 g of refined propolis, 1 g of palm pollen grains, and 0.5 g of fresh royal jelly following a very famous medicinal recipe that is extensively used as an antioxidant treatment.

### 2.2. Experimental Animals 

Twenty-four healthy adult male albino rats (120 ± 5 g) were kept in an animal room at 25 ± 2 °C and in an alternate 12 h light and dark cycle. Two weeks before the experiment was conducted, the animals were allowed to acclimatize to the testing facility conditions. Afterwards, the rats were caged equally into four experimental groups, each consisting of six rats: control (corn oil), SUM (85 mg/kg, 1/20 LD_50_ [26]), HPPJ (a mixture of 22.5 g of citrus bee honey, 1 g of refined propolis, 1 g of palm pollen grains, and 0.5 g of fresh royal jelly), and SUM-HPPJ. The rats were orally treated with 1 mL of each treatment for 28 successive days. On the second day of the last therapy, all rats were euthanized using an anesthesia system containing diethyl ether, and blood samples were gathered from the carotid artery. All animals were treated in compliance with the standard guidelines for the laboratory animal care and use protocol of the National Research Council [27].

### 2.3. Hematological Parameters

Blood samples were gently collected in anticoagulant-containing sterilized tubes (EDTA) to provide a final concentration of 5 mg/mL blood. Samples were mixed gently and discarded if any difficulties were encountered during sampling or if clots were seen in the vial. A Sysmex KX-21 hematological analyzer (Sysmex KX21, Japan) was used to evaluate the total red blood cell (RBC) count (×10^6^/µL), hemoglobin (Hb) content (g/dL), hematocrit (HCT) value (%), mean cell hemoglobin (MCH; pg), mean corpuscular volume (MCV, fL), mean corpuscular hemoglobin concentration (MCHC) values (g/dL), total white blood cell (WBC) count (×10^3^/µL), and platelet (PLT) count (×10^3^/µL) in a certified clinical laboratory.

### 2.4. Enzymatic and Biochemical Parameters 

Another set of blood samples was obtained in non-anticoagulant test tubes, allowed coagulation for 15 min at room temperature. Subsequently, they were centrifuged for serum separation for 10 min at 4000 rpm (Universal 32R, Hettich Zentrifugen model D-78532, Germany). The serum was used to examine the enzymatic and biochemical parameters. The activity of AChE was measured using the method described by Ellman et al. [28]. Blood glucose [29], uric acid [30], alkaline phosphatase (ALP) [31], total protein [32], glutathione peroxidase (GP) [33], total lipids [34], creatinine [35], glutathione-S-transferase (GST) [36], and superoxide dismutase (SOD) [37] were analyzed using BioDiagnostic kits (Diagnostic research and Reagents, Giza, Egypt). The lactate dehydrogenase (LDH) activity [38] was determined using a BioSystems Kit (BioSystems S.A., Costa Brava, Barcelona, Spain). The determination of the γ-Glutamyltransferase (γ-GT) activity was done using a Spectrum Kit (MDSS, GmbH, Schiffgraben, Germany) [39]. Each sample consisted of five replicates, and each replicate was measured three times.

### 2.5. GC-MS Analysis of the HPPJ Mixture

Approximately 2 g of HPPJ mixture was soaked overnight in 10 mL of double-distilled water. Two milliliters of the extract were then filtered using a 0.2 μm PTFE filter (Fisher Scientific, Ottawa, Canada) and a 1 mL syringe to a 1.5 mL dark high-performance liquid chromatography (HPLC) glass vial. One μL of the sample was injected into an Agilent 6890 Gas Chromatography-Mass Spectrometry (GC-MS) system supplied with an Agilent mass spectrometric detector with a direct capillary interface and HP-5 capillary column (30 m × 0.250 mm × 0.25 μm film thickness, part No. 19091J-433). Helium was used as the carrier gas at a 1 mL/min flow rate and 3 min as a solvent delay time. The mass spectrophotometric detector was performed in a state of electron impact ionization, with 70 eV energy, 50 to 800 mass/charge (*m/z*) scanning state, and at 150 °C and 230 °C as the quadrupole and ion source temperature, respectively. The voltage of electron multipliers (EM voltage) was held at 1250 V above the auto-tune. Perfluorotributylamine (PFTBA) was used to manually tune the instrument. The GC temperature system was started for 3 min at 50 °C, then increased to 280 °C at a rate of 8 °C/min and kept for 10 min. The recorded *m/z* ratio is the fingerprint of each molecule. Additionally, the separated peaks were identified using the Wiley9 and NIST14 mass spectral database. Then, the detected compounds were individually re-confirmed via online chemical databases including AMDIS, PubChem, ChemSpider, and the Chinese Chemical database.

### 2.6. Statistical Analysis

The general linear model (GLM) procedure of the Statistical Analysis System (SAS) (Version 9.3, Cary, NC, USA, 2016) was used to examine the toxicological data. Turkey’s *post-hoc* multiple comparison tests (*P* ≤ 0.05) [40] were used to compare significant means.

## 3. Results

### 3.1. Mortality and Relative Organ Weight 

No mortality was recorded among the tested animals. The relative organ weights of the kidney, liver, heart, brain, spleen, testes, and lung were presented in Table 1. The SUM-treated rats showed significantly increased brain, kidney, and liver relative weights in contrast to the control group but did not vary remarkably from the group of rats that received SUM-HPPJ. Meanwhile, the SUM treatment did not affect the relative weight of the testes while it was decreased in rats that received SUM-HPPJ. Both the control and HPPJ treatments showed similar weights.

### 3.2. Hematological Parameters

Our results showed a remarkable (*P* ≤ 0.05) decrease in RBCs, WBCs, and PLT counts, as well as hemoglobin (HGB), and MCHC contents, in SUM-intoxicated rats in comparison to the control (Table 2). The HPPJ- and SUM-HPPJ-treated rats showed similar HGB, MCV, MCH, and PLT results to those of the control group, although the SUM-treated rats had elevated MCV and MCH values (*P* ≤ 0.05) in relation to the other experimental groups. The HGB and MCHC contents of rats intoxicated with SUM were not substantially different from the control rats.

### 3.3. Serum Antioxidant Enzymes 

**Figure 1:**
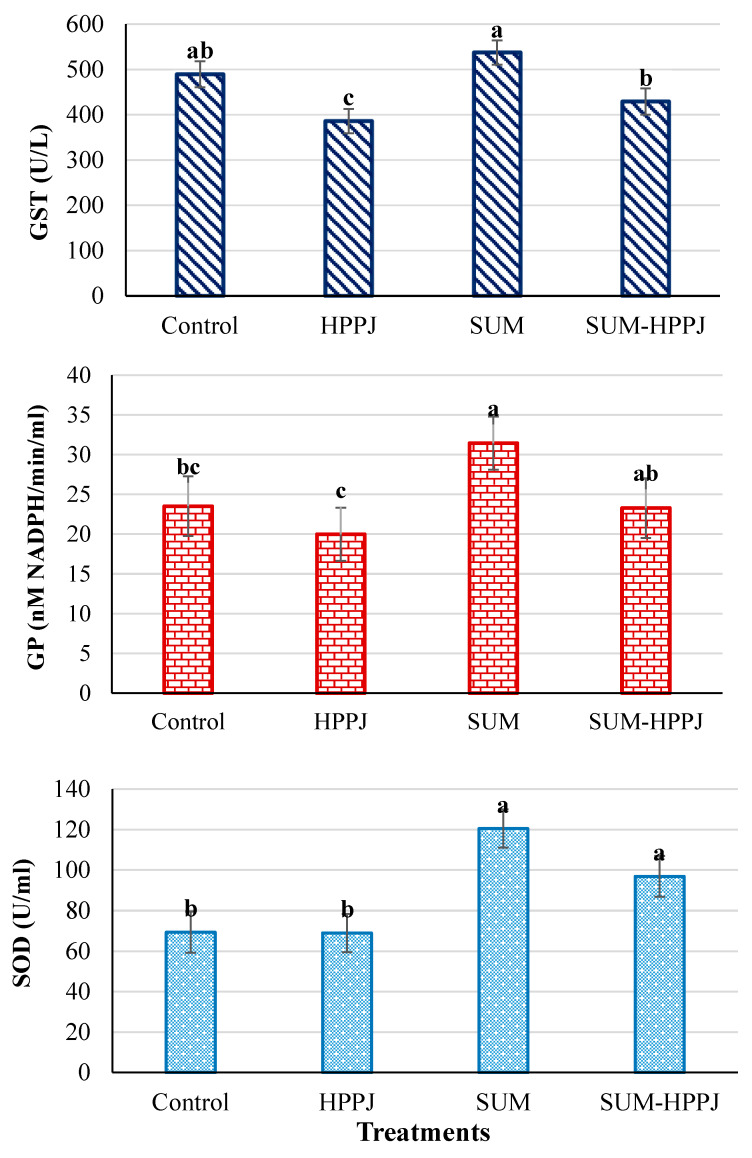
Mean activities of oxidative stress enzymes ± SE: GST; glutathione-S-transferase (U/L), GP; glutathione peroxidase (nM NADPH/min/mL), and SOD; superoxide dismutase (U/mL) of white albino rats who were orally given corn oil (Control), sumithion (SUM; 85 mg/kg, 1/20 LD_50_), HPPJ (a mixture of citrus bee honey, propolis, palm pollen grains, and royal jelly), and SUM-HPPJ for 28 consecutive days. Columns labeled with the same small letter were not significantly different based on Tukey’s *post hoc* specific comparison (*P* < 0.05).

The results (Figure 1) exhibited a remarkable (*P* ≤ 0.05) decrease in the serum GST activity (21.09%) after rats ingested HPPJ, while the SUM treatment caused an increased GST by 9.81% in relation to the control, although the SUM-HPPJ-treated rats had a GST activity relative to that of the control animals. Likewise, the GP activity in the serum samples of the SUM-treated rats increased by 33.72% in comparison to the control one, while no variations were found between SUM-HPPJ and the control. On the other hand, the GP activity decreased by 15% after rats were given HPPJ relative to the control. For the SOD activity, the SUM and SUM-HPPJ treatments increased its activity (73.72% and 39.64%, respectively), while HPPJ revealed no difference compared to the control in the serum samples.

### 3.4. Serum Hepatic Enzyme Activities

**Figure 2:**
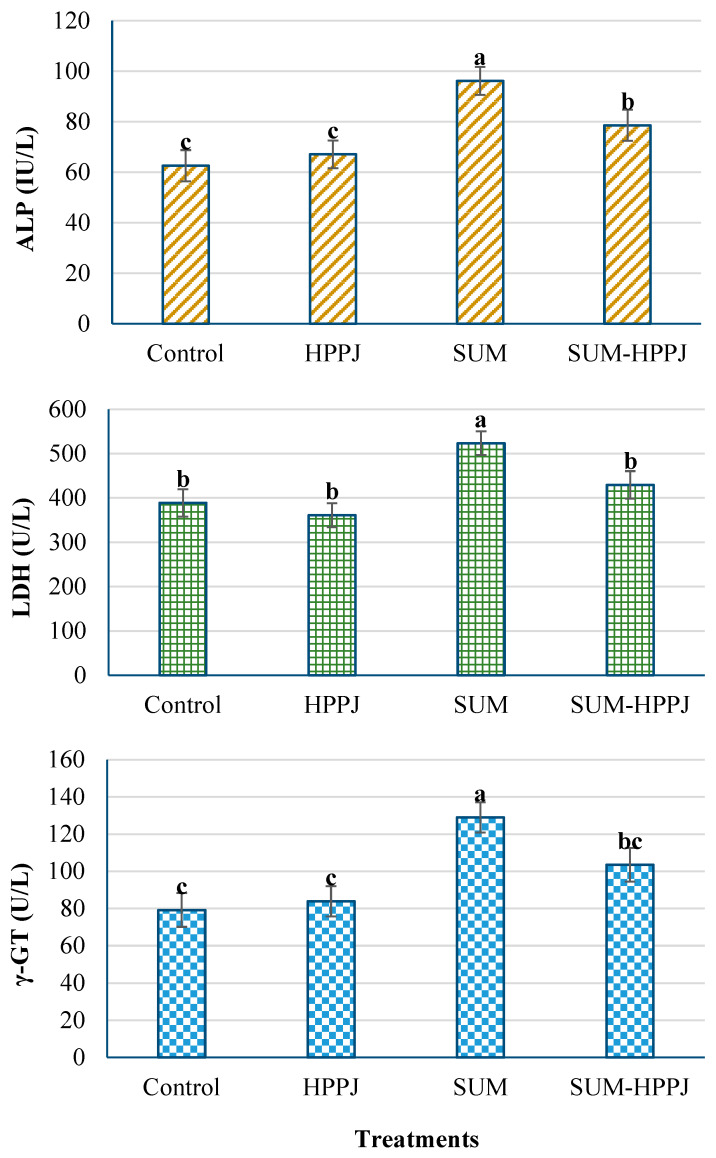
Mean activities of hepatic enzymes ± SE: ALP; alkaline phosphatase (IU/L), γ-GT; γ-glutamyl transferase (U/L), and LDH; lactate dehydrogenase (U/L) of white albino rats who were orally given corn oil (Control), sumithion (SUM; 85 mg/kg, 1/20 LD_50_), HPPJ (a mixture of citrus bee honey, propolis, palm pollen grains, and royal jelly), and SUM-HPPJ for 28 consecutive days. Columns labeled with the same small letter were not significantly different based on Tukey’s *post hoc* specific comparison (*P* < 0.05).

In the case of SUM-treated rats, a substantial increase (*P* ≤ 0.05) was found in the activity of ALP, LDH, and γ-GT by 53.6%, 34.65%, and 62.89%, respectively, compared to the control (Figure 2). The HPPJ and SUM-HPPJ treatments did not affect the ALP activity. Meanwhile, the SUM-HPPJ treatment significantly increased the activities of both LDH (10.45%) and γ-GT (30.65%) compared to the control. The HPPJ treatment had no effects on any enzymes in comparison to the control. 

### 3.5. Serum Renal and Biochemical Parameters 

The results in Table 3 disclosed that the levels of glucose, uric acid, and creatinine in SUM-treated rats increased significantly (*P* ≤ 0.05) by 27.15%, 57.34%, and 199.85%, respectively, in comparison to the control. Additionally, SUM decreased the total protein and total lipids contents and the AChE enzyme activity by about 26.53%, 57.57%, and 37.20%, respectively. However, the SUM-HPPJ-treated rats showed a reduction in the total protein, total lipids, and AChE by 13.40%, 27.44%, and 13.42%, respectively, of the control values. Creatinine increased significantly after treating the rats with SUM-HPPJ. Experimental groups treated with HPPJ showed similar values of the biochemical parameters to those of the control (*P* < 0.05), except for the protein content, which decreased. 

### 3.6. Chemical Composition of HPPJ Mixture

The HPPJ mixture was differentiated semi-quantitatively using a calculation based on chemical databases into its main chemical components using the GC-MS (Table 4). The results revealed the identification of more than 85% of the components of the HPPJ mixture as sugars, glycosides, alcohols, aldehydes, ketones, alkanes, and fatty acids. The 5-(hydroxymethyl)-furfural (HMF) was the main constituent (24.47%), followed by methyl-2-methyl-1,3-oxothiolan-2-yl-ketone (12.84%), linoleic acid (10.19%), 4H-pyran-4-one (8.57%), 4-hydroxy-3-methyl-2-butanone-1-propanol-1-D1 (5.59%), and triulose (4.32%). Furthermore, the mixture had α-d-mannofuranoside (2.81%), D-mannitol (2.28%), 2-hydroxy-2-cyclopenten-1-one (1.95%), glyceraldehyde (1.73%), propane, 1-isothiocyanato-4H-pyran-4-one (1.21%), 5-hydroxy-7-methoxy-flavone (1.07%), palmitic acid (0.79%), and D-glucitol (0.46%). Additionally, several other lactones and fatty acids were found as minor components.

## 4. Discussion

This study showed that bee products alleviated the toxicity of the organophosphorus pesticide SUM (1/20 LD_50_) in the blood, liver, and kidneys of white albino rats. It is well documented that the consumption of honey and other important bee products including royal jelly, pollen, propolis, and beebread would be valuable therapeutics [41,42]. The chemical constituents of these products are responsible for its therapeutic properties [43]. Furthermore, the inflammatory effect of SUM on organs’ weight leads to an increase in lung, liver, kidney, and heart weights, which was involved in significant injuries. Superoxide, peroxyl, hydroxyl, alcohol, hydroperoxyl, and alkoxyl radicals are the most common oxidative substances in the body, commonly referred to as ROS, created by gradual oxygen reduction and unpaired electrons that cause oxidative stress [44]. The OPs are oxidants that hinder the enzymatic antioxidant defenses such as catalase (CAT), SOD, GST, and GP [45,46,47,48,49]. On the other hand, previous research found that honey elevated the antioxidant status and resulted in a remarkable increase in the levels of packed cell volume (PCV), iron, and HGB [21,50] due to it containing phenolic acids, flavonoids, Maillard reaction products, sugars, proteins, organic acids, amino acids, and other minor compounds, which could participate in its antioxidant activity [23].

The existing study showed that SUM administration resulted in a preliminary rise in antioxidant defense mechanisms, as shown by an increase in the activity of GST and SOD enzymes. This indicated that the rats were trying to counter SUM-induced oxidative stress that caused an apparent state of oxidative stress, while the SUM group of rats that received HPPJ showed significant protection, which was indicated by the increase in the SOD, GP, and GST enzyme activity. The development of ROS and reactive intermediates following OP pesticides exposure would initiate hepatotoxicity [48,51], which causes huge damage to liver cells and function such as steatosis, chronic hepatitis, aging, inflammatory damage, and ischemic injuries [10,52,53]. The results reported herein showed that SUM affected the hepatocellular integrity. SUM induced a marked increase in serum γ-GT, LDH, and ALP. Such a rise in liver enzymes may result from hepatocellular injury and impairment in cell membranes’ permeability, resulting in the leakage of these enzymes into the bloodstream [54,55,56]. Similar findings were reported in other experiments, according to which fenitrothion has increased liver enzymes’ activity in rats [45]. The rise in ALP in SUM-treated rats may be a response to improve ALP dephosphorylation in order for additional insecticide metabolism to be excreted with bile or because of deformities in the excretion of ALP in bile by hepatocytes [57].

Honey and other bee products exhibit antioxidant activities and protective effects on the damaged liver. The HPPJ mixture caused a substantial reduction in serum ALP and γ-GT, indicating a hepatoprotective efficacy. Similar to what was reported in the current study, previous studies showed that honey has an antioxidant and hepatoprotective activity that minimizes liver damage in treated rats [58]. Additionally, injured hepatocytes lead to a decrease in the hepatic capacity to synthesize proteins [59]. Our study exhibited a substantial reduction in the total protein content in the case of rats treated with SUM in comparison to the control group, which coincides with Mossa et al. [60], who showed a reduction in the albumin content after OPs exposure, resulting from the failure in liver functions [61] and metabolism [62]. Moreover, prolonged exposure to organophosphorus pesticides causes kidney failures [63] such as chlorfenvinfos, fenitrothion, and dimethoate in rats [64]. SUM (fenitrothion) showed a decrease in creatinine clearance that could be attributed to renal damage and/or other mechanisms. Our study revealed that SUM led to an increase in serum uric acid and creatinine related to rats that received SUM-HPPJ and the control group, suggesting a kidney functional dysfunction. Our findings are consistent with other reports that confirmed that the kidney was one of the OPs target organs [47,65]. Honey has been shown to improve renal function in normal people by elevating nitric oxide levels and reducing prostaglandins [66]. Furthermore, bee pollen and honey might exert antioxidant properties and play a beneficial role in kidney function [42].

The primary effect of SUM on the exposed organisms’ nervous system is the inhibition of the AChE enzyme and an increase in the levels of acetylcholine in the cholinergic synapse. Therefore, the inhibition of butyrylcholinesterase (BuChE) and AChE is a widely known index of OPs intoxication [67,68]. In parallel with the results of the current study, cholinesterases activity inhibition may be attributed to a direct effect of fenitrothion [69] or hyperammonemia’s indirect influence, and/or a synthetic decrease in liver function due to hepatopathy [70]. Serum cholinesterases are probably produced in hepatic cells, but other organs also participate in the pool of these enzymes in plasma [71]. Thus, serum AChE activity could be a liver function indicator, and decreased enzyme activity was recorded in several liver disorders such as cirrhosis and jaundice [72,73]. However, a minimal association is recorded between organ damage and the degree of AChE inhibition induced by malathion [74,75]. Additionally, our study indicated that SUM exposure was followed by a decline in the hemoglobin level, and these findings followed those shown by Mongi et al. [76], who stated that exposure to OPs affected the hematological parameters in rats. The drop in the level of hemoglobin might be attributed to an impairment of heme biosynthesis in bone marrow [65] or might be due to OPs binding to iron, accompanied by the absence of an iron combination in hemoglobin [77]. Additionally, the current study showed decreases in the HGB concentration, platelets, RBCs, and WBCs numbers in the case of SUM-treated rats, while a portion of bee bread produced a substantial increase in the HGB, iron level, and PCV in a normal individual [21]. Similar to the ameliorative effects of the HPPJ mixture, bee propolis uses standardized PCV%, MCV, MCH, MCHC, and RBCs when used with SUM [78].

The liver is involved in the synthesis, metabolism, and transportation of lipid, as well as in impairments in the levels of plasma lipid, which may act as a simple indicator for evaluating liver dysfunctions [65]. Much research has documented changes in liver lipid profiles caused by OPs. These changes in the lipid profile may be due to fenitrothion’s effect on the hepatocyte cell membrane permeability or the bile duct blockage that decreases or prevents the secretion of cholesterol into the duodenum [45]. This may also result in the enhanced hepatic synthesis and/or decreased hepatic degradation of lipids because of a decrease in the activity of lipoprotein lipase [79]. Nagaraju et al. [48] documented dyslipidemia in rats subjected chronically to monocrotophos. Fenitrothion sub-lethal levels reversibly boosted triglycerides in mice after 90 days of exposure [53]. The current findings revealed that administering fenitrothion induced homeostasis of glucose, as confirmed by a substantial gradual rise in the blood glucose level. Furthermore, previous research suggested that repeated exposure to OPs causes hyperglycemia and glycogen depletion in the muscle and liver [48,65]. This may be due to the impaired transport of glucose and the synthesis of glycogen [80]. Besides, elevated blood glucose levels can result from an imbalance between hepatic glucose output and peripheral glucose uptake [81], or as a result of an unexpectedly increased catabolism to meet higher energy requirements caused by OPs [82]. Animal studies indicate that glycogen phosphorylase activity, an enzyme that splits glycogen into glucose and decreases the hepatic glycogen content, improves following exposure to fenitrothion and malathion [53,83]. This was confirmed by the reduction in the concentration of hepatic glycogen after SUM exposure in fish [84]. Honey and bee products have demonstrated antidiabetic effects from animal models to clinical studies [21,85]. Correspondingly, the HPPJ-treated rats displayed a substantial reduction in glucose levels compared to control and rats treated with SUM. Researchers addressed this as a possible antidiabetic agent, and this effect is related to the existence of fructose [86]. Another possible mechanism illustrates the hypoglycemic effect of honey through the modulation of the insulin signaling pathway [87].

The protective activity of this novel medicinal recipe of honey and bee products used in the current study was not reported elsewhere (to the best of our knowledge) and might be due to its content of sugars, glycosides, alcohols, aldehydes, ketones, alkanes, and fatty acids. The HMF attenuated liver damage (fibrosis) after CCl_4_ [88] and alcohol-induced liver damage [89], and functioned as a cardioprotective agent [90]. Furthermore, the various components of ketones, linoleic acid, 4H-pyran-4-one, triulose, α-d-mannofuranoside, D-mannitol, 2-hydroxy-2-cyclopenten-1-one, glyceraldehyde, propane, 1-isothiocyanato-4H-pyran-4-one, 5-hydroxy-7-methoxy-flavone, palmitic acid, and D-glucitol were reported as having antioxidant activity [19,23,50,91].

## 5. Conclusions

Our findings indicate that SUM caused remarkable damage to the kidney and liver, resulting in imbalances in their functions. Strikingly, the liver was the first organ affected by SUM uptake and may delayed the impact of SUM on the kidney due to pharmacokinetic conditions or to deficiencies in hepatic production of essential proteins, which allowed the weakened liver to affect other organs. It also caused harmful ROS production which produced oxidative stress. Oxidative stress conditions affected the weights of the kidney, body, and liver and changed their biochemical indicators. The present study was motivated by the possible health benefits of bee products including royal jelly, honey, and propolis, in addition to palm pollen grains. These products are extremely abundant in active ingredients, including phenolic compounds, phenolic acid, flavonoids, enzymes, and terpenes, which have biological roles in preventing several disorders and promoting good health. The present study revealed the preventive effect of honey and other bee products against liver injury, and a synergistic effect may be considered. The mechanism of action may be linked to the bee product’s antioxidant and anti-inflammatory function.

## Figures and Tables

**Table 1:** The mean relative organ weights ± SE of the liver, lung, kidney, heart, brain, spleen, lung, and testes of white albino rats who were orally administered corn oil (Control), sumithion (SUM; 85 mg/kg, 1/20 LD_50_), HPPJ (a mixture of citrus bee honey, propolis, palm pollen grains, and royal jelly), and SUM-HPPJ for 28 consecutive days.

Group	Liver ± SE	Kidney ± SE	Heart ± SE	Brain ± SE	Spleen ± SE	Lung ± SE	Testes ± SE
**Control**	3.11^c^ ± 0.105	0.73^bc^ ± 0.042	0.38^a^ ± 0.026	0.713^b^ ± 0.036	0.27^a^ ± 0.059	0.73^a^ ± 0.045	1.13^a^ ± 0.062
**HPPJ**	3.36^bc^ ± 0.105	0.60^c^ ± 0.042	0.34^a^ ± 0.026	0.704^b^ ± 0.032	0.33^a^ ± 0.046	0.64^a^ ± 0.045	1.17^a^ ± 0.062
**SUM**	3.64^ab^ ± 0.105	0.82^ab^ ± 0.042	0.40^a^ ± 0.023	0.928^a^ ± 0.032	0.26^a^ ± 0.046	0.76^a^ ± 0.040	1.06^a^ ± 0.062
**SUM-HPPJ**	3.99^a^ ± 0.122	0.94^a^ ± 0.042	0.44^a^ ± 0.026	0.932^a^ ± 0.036	0.24^a^ ± 0.051	0.79^a^ ± 0.045	0.76^b^ ± 0.071

n = 6 rats/group, SE; standard error, means with the same superscript letter were not significantly different, *P* ≤ 0.05, and relative organ weight = (organ weight/final body weight) × 100.

**Table 2:** Mean ± SE values of the total white blood cell counts (WBC; × 10^3^/µL), total red blood cell count (RBC; × 10^6^/µL), hemoglobin content (HGB; g/dL), hematocrit value (HCT; %), mean corpuscular volume (MCV; fL), mean cell hemoglobin (MCH; pg), mean corpuscular hemoglobin concentration (MCHC; g/dL), and platelets number (PLT; × 10^3^/µL) of white albino rats who were orally given corn oil (Control), sumithion (SUM; 85 mg/kg, 1/20 LD_50_), HPPJ (a mixture of citrus bee honey, propolis, palm pollen grains, and royal jelly), and SUM-HPPJ for 28 consecutive days.

Group	WBC ± SE	RBC ± SE	HGB ± SE	HCT ± SE	MCV ± SE	MCH ± SE	MCHC ± SE	PLT ± SE
**Control**	11.20^a^ ± 0.285	5.54^ab^ ± 0.188	10.78^ab^ ± 0.198	34.28^a^ ± 0.980	59.47^b^ ± 2.530	18.68^b^ ± 0.716	31.43^b^ ± 0.428	566.8^a^ ± 38.81
**HPPJ**	10.33^ab^ ± 0.247	6.05^a^ ± 0.146	11.08^a^ ± 0.177	34.24^a^ ± 0.877	56.58^b^ ± 2.263	18.32^b^ ± 0.640	32.37^ab^ ± 0.383	579.2^a^ ± 34.71
**SUM**	7.73^c^ ± 0.247	3.96^c^ ± 0.146	9.98^b^ ± 0.198	31.94^a^ ± 0.877	81.10^a^ ± 2.263	25.79^a^ ± 0.640	31.85^b^ ± 0.383	253.8^b^ ± 34.71
**SUM-HPPJ**	9.87^b^ ± 0.285	5.28^b^ ± 0.163	10.50^ab^ ± 0.198	31.25^a^ ± 0.980	59.50^b^ ± 2.530	20.00^b^ ± 0.716	33.63^a^ ± 0.428	522.5^a^ ± 38.81

Means were statistically compared using the Tukey’s *post-hoc* multiple comparison methods at *P* ≤ 0.05. Means with the same superscript letter were not significantly different. Each treatment was replicated six times, and each replicate was measured three times.

**Table 3:** Mean ± SE values of glucose (mg/dL), total protein (g/dL), total lipids (mg/dL), uric acid (mg/dL), and creatinine (mg/dL) contents and acetylcholine esterase (AChE; nM ATChI/min) activity of white albino rats who were orally given corn oil (Control), sumithion (SUM; 85 mg/kg, 1/20 LD_50_), HPPJ (a mixture of citrus bee honey, propolis, palm pollen grains, and royal jelly), and SUM-HPPJ for 28 consecutive days.

Group	Glucose ± SE	Total Protein ± SE	Total Lipids ± SE	Uric Acid ± SE	Creatinine ± SE	AChE ± SE
**Control**	60.80^b^ ± 5.462	7.54^a^ ± 0.477	229.75^a^ ± 13.795	2.93^b^ ± 0.255	0.667^c^ ± 0.094	20.94^a^ ± 0.662
**HPPJ**	62.39^b^ ± 4.885	6.60^b^ ± 0.427	211.90^a^ ± 12.338	3.16^b^ ± 0.228	0.859^c^ ± 0.084	20.23^a^ ± 0.592
**SUM**	77.31^a^ ± 4.885	5.54^c^ ± 0.427	97.47^c^ ± 12.338	4.61^a^ ± 0.228	2.000^a^ ± 0.084	13.15^c^ ± 0.592
**SUM-HPPJ**	67.50^b^ ± 5.462	6.53^b^ ± 0.477	166.71^b^ ± 13.795	3.91^ab^ ± 0.255	1.078^b^ ± 0.094	18.13^b^ ± 0.662

Means were statistically compared using the Tukey’s *post-hoc* multiple comparison methods at *P* ≤ 0.05. Means with the same superscript letter were not significantly different. Each treatment was replicated six times, and each replicate was measured three times.

**Table 4:** List and percentages of chemical components of the citrus bee honey, refined propolis, palm pollen grains, and royal jelly mixture that was analyzed using gas chromatography-mass spectrometry (GC-MS) and identified from the mass spectrum of each molecule using the Wiley9 and NIST14 mass spectral databases. Then, individually listed compounds were re-confirmed through the online databases AMDIS, PubChem, ChemSpider, and Chinese Chemical database.

Chemical Component	% in the Extract of Total
HMF	24.47
Ketone, methyl-2-methyl-1,3-oxothiolan-2-yl	12.84
Linoleic acid (9,12-Octadecadienoic acid)	10.19
4H-Pyran-4-one	8.57
4-Hydroxy-3-methyl-2-butanone1-Propanol-1-D1	5.59
Triulose	4.32
α-d-Mannofuranoside	2.81
N1, N1-Dimethyl-N2-n-butylformamidine	2.58
D-Mannitol	2.28
2-Hydroxy-2-cyclopenten-1-one	1.95
Glyceraldehyde	1.73
Propane, 1-isothiocyanato-4H-pyran-4-one	1.21
Lanosta-7,9(11)-diene-3, beta-18,20-triol-3,18-diacetate	1.16
Flavone, 5-hydroxy-7-methoxy	1.07
Distannoxane, hexabutyl	0.86
Palmitic acid	0.79
D-Glucitol	0.46
α-D-Galactopyranose	0.44
d-Glycero-d-heptose	0.29
3-Deoxy-d-mannoic lactone	0.28
2,5-Dihydroxy-3,6-dimethylhydroxy-1,4-dioxane	0.18
Heptadecanoic acid	0.13
